# Field implementation using chlorophyll derivatives with sunlight for malaria, filaria and dengue fever vectors control in infested Africa swamps

**DOI:** 10.1186/1475-2875-11-S1-P42

**Published:** 2012-10-15

**Authors:** Mahmoud H Abdel-Kader, Tarek A El-Tayeb

**Affiliations:** 1 Faculty of Pharmacy and Biotechnology, German University in Cairo, New Cairo, Egypt; 2 National institute of Laser Enhanced Sciences (NILES), Cairo University, Cairo Egypt

## 

In this work, we present the successful field implementation of using Photodynamic modality to control Malaria, Filaria and Dengue Fever vectors in infested epidemic swamps in Uganda, Ethiopia and Sudan. As the Photodynamic technique has become a major approach for the control of human parasites and noxious insects.

Field investigations were carried out based on laboratory and semi-field results. In these trials, chlorophyll derivatives were added to the infested swamps to be taken by the mosquito larvae and the accumulated photoactive compound (photosensitizer) inside the larvae body induces upon sunlight exposure an oxidation stress, that results in organism death.

As example in Kasangati and Namanve cities of Wakiso a district in Uganda , chlorophyll derivatives , as sunlight active photosensitizers was applied to cover 250 000 square meter of infected swamps and sand pits (4 gm/ m^2^).The infected cities were mapped for this field study using Geographical Positioning System (GPS). All the biotic and a-biotic factors were measured before and after treatment. Confocal laser scanning microscopy measurements were used to monitor the concentration and the dynamic distribution of chlorophyll derivatives inside the *larvae of Anopheles gambiae* mosquito.

The results reveal that from 85% to 100% mortality of larvae population are obtained at different concentrations of Chlorophyll derivatives (0.1 -100µM). Other biological beneficiary organisms, such as the dragon fly larvae and mosquito predator larvae, which were present in the same treated swamps were not affected (target selectivity).

The field trials are the result of three years continuous and persistent work, which showed promising success in controlling Malaria, Filaria and Dengue Fever vectors by cutting the mosquito’s life cycle without new generation, formation, or reinfestation.

